# Localized Refractive Changes Induced by Symmetric and Progressive Asymmetric Intracorneal Ring Segments Assessed with a 3D Finite-Element Model

**DOI:** 10.3390/bioengineering10091014

**Published:** 2023-08-27

**Authors:** Gonzalo García de Oteyza, Juan Álvarez de Toledo, Rafael I. Barraquer, Sabine Kling

**Affiliations:** 1 Clínica Oftalmológica García de Oteyza, 08017 Barcelona, Spain; gonzalo_gdeoteyza@hotmail.com; 2 Escuela de Doctorado, Universidad Autónoma de Barcelona (UAB), 08193 Barcelona, Spain; 3 Oftalvist Barcelona, 08017 Barcelona, Spain; 4 Centro de Oftalmología Barraquer, 08021 Barcelona, Spain; 5 Universitat Internacional de Catalunya (UIC), 08017 Barcelona, Spain; 6 Institute for Biomedical Engineering, Department of Information Technology and Electrical Engineering, ETH Zurich, 8092 Zurich, Switzerland; 7 ARTORG Center for Biomedical Engineering Research, University of Bern, 3010 Bern, Switzerland

**Keywords:** finite element model, keratoconus, refractive surgery, corneal biomechanics, elastography, 3D model

## Abstract

To build a representative 3D finite element model (FEM) for intracorneal ring segment (ICRS) implantation and to investigate localized optical changes induced by different ICRS geometries, a hyperelastic shell FEM was developed to compare the effect of symmetric and progressive asymmetric ICRS designs in a generic healthy and asymmetric keratoconic (KC) cornea. The resulting deformed geometry was assessed in terms of average curvature via a biconic fit, sagittal curvature (K), and optical aberrations via Zernike polynomials. The sagittal curvature map showed a locally restricted flattening interior to the ring (Kmax −11 to −25 dpt) and, in the KC cornea, an additional local steepening on the opposite half of the cornea (Kmax up to +1.9 dpt). Considering the optical aberrations present in the model of the KC cornea, the progressive ICRS corrected vertical coma (−3.42 vs. −3.13 µm); horizontal coma (−0.67 vs. 0.36 µm); and defocus (2.90 vs. 2.75 µm), oblique trefoil (−0.54 vs. −0.08 µm), and oblique secondary astigmatism (0.48 vs. −0.09 µm) aberrations stronger than the symmetric ICRS. Customized ICRS designs inspired by the underlying KC phenotype have the potential to achieve more tailored refractive corrections, particularly in asymmetric keratoconus patterns.

## 1. Introduction

Biomechanical simulations describe the response of a biological material to an applied load in terms of a numerical expression and relate different input parameters (material properties and applied forces) to an output (deformed shape) [1] based on the internal constitution of the material. The aim of modelling the human corneal tissues is to predict the refractive results or compare different surgical interventions. For this purpose, the tissue must be described by a model that incorporates anatomically related information, which needs to be modified in the case of pathological situations [2]. In the past, finite element modelling (FEM) has been demonstrated successful in modelling keratoconic corneas [3]. Keratoconus is a type of degradative localized corneal weakening, which manifests in a conical outbuilding, irregular astigmatism, increased optical aberrations and consequently in, a relevant loss of visual acuity. Corneal modelling has improved in refinement and precision from a single-membrane model with linear elastic [4] or viscoelastic [5] material properties to hyperelastic orthotropic shell models that account for the tissue’s microstructure [6], as well as solid viscoelastic and nonlinear [7] corneal models that account for time-dependent effects and large deformations, up to patient-specific models [3]. Yet, previous literature on the implantation of intracorneal ring segments (ICRSs)—small arc-shaped PMMA (polymethyl methacrylate) implants of different thicknesses and arc lengths, which are implanted into the keratoconic cornea aiming to alter its geometry and improve its refractive properties and patient’s visual acuity—is limited. Initial studies concentrated on axisymmetric models to evaluate the effect of different ICRS geometries on the induced refractive change [8]. More recently, three-dimensional models considering a generic [9] or patient-specific cornea [10] have been proposed, having the potential of becoming more clinically relevant, yet still cannot capture the post-operative remodelling effects and are much more computationally expensive. Therefore, currently, the greatest value of FEM and ICRS implantation seems to be the comparison of different implant designs.

Since the recent phenotypic keratoconus (KC) classification described by Alfonso [11], in which the corneal curvature pattern becomes the most relevant factor in the surgical management of the ectasia, the understanding of the refractive outcome after intracorneal ring segment (ICRS) implantation has evolved considerably. So far, all kinds of phenotypes have been treated with symmetric ICRSs. Unfortunately, as a result, in some cases, topographic and comatic outcomes and patient satisfaction after surgery were poor and disappointing [12]. It is speculated that the reason for poor results is that ICRSs do not address the asymmetry present in the large majority of KC corneas [12]. Therefore, symmetric ICRSs have been considered excellent in the astigmatic correction of symmetric phenotypes but deficient in the control of coma and corneal irregularity in asymmetric patterns, which could be fundamental to achieving better visual results [12]. This hypothesis has led the scientific community to investigate different ring segment design variations, including progressive asymmetric ICRSs [13,14,15,16].

The current study aims to develop a three-dimensional (3D) finite element model to evaluate the theoretically expected local geometrical effects of a new progressive intrastromal ring segment with varying ring thicknesses and base widths in a healthy and a keratoconic in silico model. Special attention will be paid to the analysis of localized curvature changes and optical aberrations. The model shall be evaluated both in a generic healthy and a generic keratoconic cornea to better evaluate the impact of localized tissue weakening in disease.

## 2. Materials and Methods

### 2.1. Finite Element Modelling

A FEM was created in ANSYS software (Mechanical APDL, Release 19.2, Canonsburg, PA, USA), consisting of the three-dimensional anterior half of the eye coat. The aspheric corneal button was attached to the sclera, and the latter allowed sliding radially at the eye equator. Corneal tissue was modelled as two shells representing the anterior and posterior stroma above and below the implantation depth. A joint corneal thickness of 550 µm was assumed. A quadrilateral mesh consisting of 23,232 elements of SHELL181 (a 4-node structural shell element) was created, and hyperelastic isotropic material properties (Yeoh) were assigned to the stroma and sclera, similar to previously [9]. Isotropic elastic material properties were defined for the material of the ICRS implant (PMMA). All modeling-related details are summarized in Table 1.

**Figure 1:**
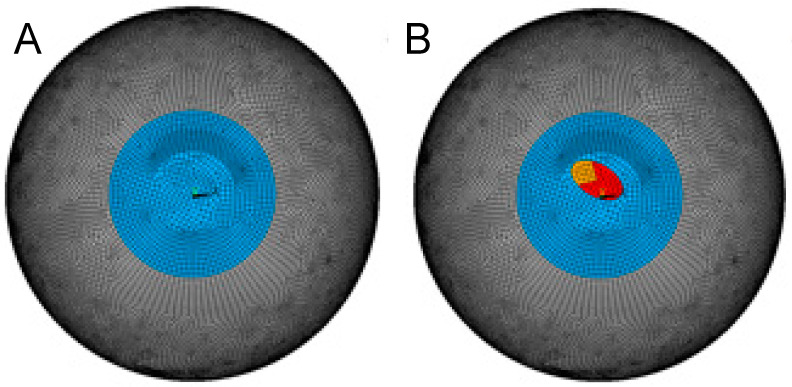
Meshed geometry of the healthy (**A**) and keratoconic (**B**) corneal model.

Isotropic material properties (i.e., not considering fibril orientation) were considered a reasonable approximation for both the healthy and keratoconic region of the cornea because (i) a similar refractive response after ICRS implantation is observed in human and porcine corneas, even though the two species have different predominant fibril orientations [17], suggesting that collagen ultrastructure plays a negligible role. (ii) In keratoconus, fibril orientation is lost [18] due to structural degeneration. Contact elements were defined between the anterior and posterior shell to model the intact, healthy cornea. The stress-free geometry was inversely determined such that after simulating normal intraocular pressure (IOP) by applying a surface pressure of 15 mmHg to the posterior surface, the refractive power of the anterior stressed cornea was approx. 43 diopters. Simulations were conducted for a healthy and a keratoconic cornea. The latter was modelled by assigning a locally weakened region with a reduced stiffness of 30% and 70% in the more central and lateral regions (Figure 1), respectively. The size and shape of the weakened zone roughly correspond to the dimensions and degree of shear modulus weakening reported previously [19]. This mechanical modification resulted in approximately the same topographic change (sagittal curvature map) as in a grade 4 keratoconus in the current study.

Tunnel creation and ICRS insertion were simulated by deleting contact elements between the anterior and posterior shell elements and by defining new contact elements between the top and bottom ICRS surfaces with the tunnel, respectively. The tunnel dimensions were simulated to be 10% larger than the typical dimension used for femtosecond laser creation (5.8 and 7.1 mm inner and outer diameter, respectively). This allowed us to account for the tunnel expansion induced during manual ring insertion, which is a natural consequence as the circumference of the tunnel is smaller than that of the ICRS (13.0 mm vs. up to 18 mm for the 800 µm wide and 300 µm high segment). We may expect that the tissue at the edges of the tunnel experiences the highest tensile load in radial direction and that subjecting corneal tissue to tensile stress oriented orthogonally to the fibre orientation likely facilitates tissue rupture and expands the stromal tunnel in the real clinical situation.

One of the ring segments considered in the simulation is the progressive, asymmetric ICRS fabricated by AJL (AJL Ophthalmic S.A., Vitoria, Álava, Spain) that spans over an arc length of 160° and has a triangular cross-section and an optical zone of 6 mm. Its thickness increases from 150 to 300 µm, and its base width increases from 600 to 800 µm from one end to the other. The ICRS geometry was meshed with triangular SOLID185 elements (an 8-node structural solid element). Isotropic material properties corresponding to PMMA were assigned (E = 3300 MPa, Poisson’s ratio = 0.4) to the implant [20]. In addition to the original ICRS geometry, we also evaluated the effect of only thickness variation (base width 700 µm, thickness 150 to 300 µm, *asymTH*) and only width variation (base width 600 to 800 µm, thickness 225 µm, *asymW*) as a reduced form of asymmetric ICRS design.

For comparison with a standard ICRS, we considered a symmetric ICRS (*sym*) with constant thickness (225 µm) and constant base width (700 µm). While this geometry does not actually exist on the market, it has the advantage that it represents the dimensions of the asymmetric ICRS at 80°, i.e., at half the arc length. The closest commercially available symmetric ICRS with the same optical zone might be Keraring, with 200 or 250 µm thickness and a base width of 800 µm. In addition to this hypothetical ICRS, the maximal (300 µm thickness, 800 µm base width, *symMax*) and minimal (150 µm thickness, 600 µm base width, *symMin*) cross-section of the asymmetric ICRS were simulated as a symmetric ICRS for better theoretical interpretation. Only one of these extreme dimensions actually exists on the market (150/600), but it is usually employed in optical zones of 5 mm in central phenotypes.

### 2.2. Curvature Analysis

For average evaluation of corneal curvature, coordinates of the deformed geometry were exported from ANSYS and read into Matlab (version R2017b, The MathWorks Inc., Natick, MA, USA). Then, a bi-conic surface was fitted according to

(1) $$S_{ant}=\frac{\frac{x^{2}}{R_{x}}+\frac{y^{2}}{R_{y}}}{1+\sqrt{1-\left(1+Q_{x}\right)\cdot \frac{x^{2}}{R_{x}^{2}}-\left(1+Q_{y}\right)\cdot \frac{y^{2}}{R_{y}^{2}}}}$$

where *S*_ant_ represents the detected corneal elevation, and x and y are the corresponding coordinates. *R*_x_ and *R*_y_ are the two radii of curvature in the direction of x and y, and *Q*_x_ and *Q*_y_ are the corresponding q-values describing asphericity. In addition, the maximal vertical displacement compared to the IOP-only condition was determined as an approximation of the induced change in axial length ∆AL of the eye. *R*_x_ and *R*_y_ were subsequently converted into refractive power dpt by

dpt = (*n*_0_ − *n*_1_)/*R* (2)

where *n* is the refractive index with values of *n*_0_ = 1 and *n*_1_ = 1.375 for the anterior surface and *n*_0_ = 1.375 and *n*_1_ = 1.333 for the posterior surface; *R* is the corresponding radius of curvature within an optical zone of 5 mm diameter.

For local evaluation of corneal curvature, sagittal curvature maps and difference maps were computed for the IOP-only and ICRS-implanted corneas. To calculate sagittal curvature in the keratoconic model, first, the most elevated point on the corneal surface was determined and subsequently defined as the origin of coordinates. In the healthy corneal model, the apex and the origin of coordinates coincided.

### 2.3. Optical Aberrations

Sixth-order Zernike polynomials were fitted to the topography data of the anterior cornea for a detailed analysis of optical aberrations. A custom routine written in Matlab was used for this purpose. Results are presented adhering to the OSA standard indices. For interpretation, only fourth-order terms were considered (with a particular interest in spherical aberration), given that fifth-order and higher-order terms are mostly irrelevant in clinical patients, including keratoconic ones.

## 3. Results

### 3.1. Average Anterior Corneal Curvature

In the healthy cornea, the asymmetric ICRS induced a slightly lower corneal flattening compared to the mean symmetric ICRS (−2.8 versus −3.9 dpt), while the maximal change in axial length was similar in both ICRS types (37 versus 36 µm). With an assumed total ocular refractive power (cornea + lens) of 60 diopters [21], the change in axial length of 36 to 37 µm corresponds to a myopic shift of 0.13 diopters. This means the effective refractive change was −2.9 and −4.1 dpt in the asymmetric and symmetric ICRSs, which falls in the range of clinical outcomes [13]. As expected, the induced curvature changes and axial length reduction were largest with the maximal symmetric ICRS (−4.8 dpt) and smallest with the minimal symmetric ICRS (−2.5 dpt). Interestingly, thickness variation or base width variation alone achieved a substantially higher curvature change (both −4.1 dpt) than both in combination (−2.9 dpt).

The simulation of keratoconus resulted in corneal steepening, particularly in the horizontal meridian, with an average increase in the ocular refractive power by +4.7 dpt. Similar to the healthy cornea, in the keratoconic cornea, the asymmetric ICRS induced a lower flattening compared to the symmetric ICRS (−0.6 versus −1.9 dpt). The reduction in axial length and corresponding myopic shift was similar in both ICRS types (0.16 diopters). Compared to the maximal and minimal symmetric ICRS, again, the overall corneal flattening was largest with the maximal symmetric ICRS (−3.9 dpt) and smallest (even contrary) with the minimal ICRS (+2.4 dpt). In the keratoconic cornea, width variation only induced a stronger flattening (−2.1 dpt) and height variation only a lesser flattening (−0.3 dpt) than both the asymmetric and average symmetric ICRSs. Table 2 presents values for the different radii of curvature, their changes, and the reduction in axial length and q-values for the different ICRS designs.

### 3.2. Local Curvature

**Figure 2:**
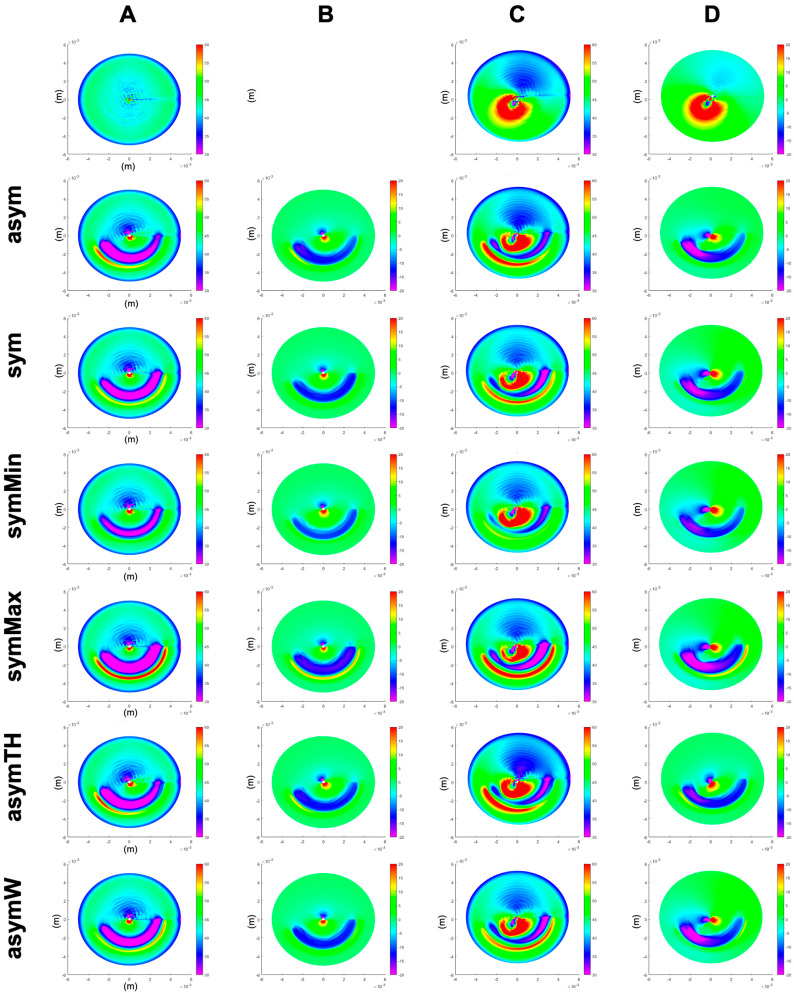
Sagittal curvature (**A**,**C**) and differential maps after simulated ICRS implantation (**B**,**D**) in a generic healthy (**A**,**B**) and a generic keratoconus (**C**,**D**) cornea. First row: the pre-op healthy (**A**) and keratoconus (**C**) cornea, as well as their difference (**D**). asym = progressive asymmetric ICRS, sym = average symmetric ICRS, symMin and symMax = symmetric ICRS with the minimal and maximal cross-section of the progressive asymmetric ICRS, asymTH = asymmetric ICRS with thickness-only variation, and asymW = asymmetric ICRS with width-only variation.

Figure 2 presents the refractive curvature maps as well as the changes in corneal elevation (each with respect to the stressed pre-op corneal shape) for a healthy and keratoconic cornea, respectively. The induced flattening pattern in the sagittal curvature map is conceptually similar to clinical data, in particular, the pattern of localized restricted flattening within the region enclosed by the ring and localized steepening on the opposite half of the cornea. However, it is evident that the model cannot account for post-operative epithelial remodelling after ICRS implantation, preventing the analysis of corneal surface homogenization in the current study.

### 3.3. Optical Aberrations

Table 3 presents the induced optical aberrations in terms of values for Zernike coefficients retrieved from the polynomial fit. Looking at the pre-op keratoconic cornea, the disease primarily manifested in vertical coma (+5.29 µm), defocus (−4.64 µm), oblique primary and secondary astigmatism (2.60 and −2.49 µm), horizontal coma (+2.37 µm), primary spherical aberration (+1.68 µm), negative vertical secondary astigmatism (+1.63 µm), vertical/horizontal primary astigmatism (+1.61 µm), and oblique trefoil (+1.42 µm). All ICRS designs corrected defocusing, vertical/horizontal primary astigmatism, and vertical secondary astigmatism terms by inducing the opposite aberration. In the healthy eye, the asymmetric and the mean symmetric ICRS induced a similar extent of vertical coma (−1.89 vs. −1.81 µm) and defocus (1.18 vs. 1.21 µm) aberrations. In the keratoconic cornea, the induced correction of vertical coma (−3.42 vs. −3.13 µm) and defocus aberrations (2.90 vs. 2.75 µm) was overall more pronounced than in the healthy cornea and slightly higher with the progressive design. The progressive and the thickness variation-only ICRS induced a correction of horizontal coma (−0.67 and −0.62 µm), while the remaining ICRS designs increased this type of aberration (range 0.00 to +0.44 µm). Similarly, the progressive and the thickness variation-only ICRS also corrected the oblique trefoil (−0.54 and −0.71 µm) and the oblique secondary astigmatism (+0.48 and +0.50 µm) aberrations best.

## 4. Discussion

The presented three-dimensional FEM of ICRS implantation further expands on our previous two-dimensional simulation study, which predicted a more tailored refractive correction of asymmetric keratoconic patterns with a progressive asymmetric ICRS design [22]. In the former 2D study, a progressive asymmetric ICRS was found to be advantageous for two reasons: first, it provokes a more remarkable refractive change near the region of the cone. Second, by varying both base width and ring thickness, the refractive change responds to the different degrees of required flattening at different angular positions. Thus, this type of ICRS is expected to produce a less distorted central optical zone. It should be maximally effective, positioning the higher and thicker part of the ICRS into the steepest area of the cone.

Our current results confirm previous findings and demonstrate the third advantage of a progressive asymmetric ICRS, which may be the most relevant to understanding the benefits of these kinds of rings in asymmetric KC phenotypes. Namely, an asymmetric ICRS achieves a more balanced correction of coma, primary astigmatism, defocus, and trefoil aberrations. Prominently, the correction of horizontal coma was highest with the asymmetric ICRS, even when comparing the refractive outcome with a hypothetical symmetric ICRS that adopted either the cross-section of the smallest or largest end of the asymmetric ICRS. Our results indicate that thickness variation, in particular, addresses horizontal coma, oblique trefoil, and oblique secondary astigmatism. Nevertheless, differences observed in the induced refractive changes in the generic healthy and keratoconus cornea by the same ICRS emphasize the importance of the underlying corneal geometry and thus of the keratoconus phenotype on the achieved outcome.

Quality of vision is recognized as one of the most essential parameters in keratoconus patients. Primary coma and high-order aberrations are the main optical aberrations degrading visual quality in keratoconus [23,24]. So far, aberrometric findings have hardly been considered in clinics to interpret the outcome after ICRS implantation. In keratoconus patients with no coincident topographic and comatic axes, Alfonso et al. found a decrease in coma-like RMS from 0.80 ± 0.53 μm before surgery to 0.61 ± 0.59 μm for a 4.5 mm pupil [25] after implanting a constant and symmetric ICRS. Vega-Estrada and colleagues reported a lower change from 4.12 to 3.55 μm with an asymmetric ICRS (different design), suggesting that further design optimizations were necessary [16]. Al-Tuwairqui [15] compared a 360° ring (Myoring) and a symmetric ring segment (Keraring). This group found that only with the 360° ring coma improved significantly (by 27.4%). In contrast, in a recent clinical study applying the same asymmetric ICRS as in the current study, primary coma was decreased [26] by 40.1% (*p* < 0.001), confirming the remarkable efficacy of this design of ring segment to reduce this type of aberration, which, according to Piñero, has demonstrated to have a very negative impact on visual acuity in keratoconus [12] patients.

It is also important to interpret the results of the current simulation study regarding corneal flattening in the context of the phenotype studied in this simulation. The “duck” phenotype is characterized by an overall oval conical convex appearance, in which the largest part of the cone is situated inferiorly to the corneal apex but a small part reaches into the superior peripheral cornea. Moreover, the “duck” morphology can be defined as well by a non-coincidence between the comatic and topographic axes in a low asphericity paracentral phenotype. This difference among axes should be greater than 30° and lower than 60°. Due to the generic nature of our model, the “duck” phenotype could not be fully reproduced. In further studies, this kind of asymmetric phenotype should be studied since it is in those precise cases where asymmetric ICRSs are particularly recommended. We hypothesize that both average and local curvature could obtain even better results if a “duck” phenotype was achieved in the model. In fact, there are two papers that study the impact of ICRS in the “duck” phenotype. Alfonso et al. studied the symmetric ICRS [25], and Kammoun et al. used an asymmetric ICRS, the same as modelled in our current study [26]. In their study, Alfonso observed an overall reduction in sphere, cylinder, and spherical equivalent in most of the patients, but the results did not reach statistical significance. Kammoun et al. later confirmed these clinical results, this time with statistical significance. It is worth noting that coma decreased by 25% with a symmetric ICRS in the “duck” phenotype” and by 42% with an asymmetric ICRS. With the symmetric ICRS, five eyes lost at least one line of vision, which is likely to happen when aberrations are not well corrected.

A limitation of the current study is that, similar to previous simulation studies, the refractive changes predicted by the model overestimated actual clinically achieved refractive outcomes, particularly with respect to the sagittal curvature maps. Nevertheless, the simulated sagittal curvature map agrees well with the immediate post-operative effects observed experimentally in ex vivo porcine eyes [27], indicating that dynamic changes occurring during the post-op time play a role that has not been considered by the model. These changes include viscoelastic material deformation, as well as ongoing tissue remodelling after surgery (including both epithelium and stroma) that leads to a local adaptation of surrounding tissue, attenuating the initially induced pure geometrical response. The fact that tissue remodelling is currently not well described and thus hard to implement in simulations further complicates the prediction of refractive changes with ICRS implantation. Remodeling effects will, however, only smooth and not completely change the actual pattern of induced curvature changes, which was the principal interest of this study. This way, the current study helps identify differences in the underlying optomechanical mechanism of different ICRS designs. A further limitation inherent to the current study includes the assumption of a homogenous hyperelastic material for the stroma. It has been described earlier that the corneal microstructure has a low impact on the deformation response provoked by an ICRS, hence justifying the use of a homogenous solid [28]. Future simulation studies on ICRS implantation are desired to include patient-specific elevation maps and more refined material models.

## 5. Conclusions

The present study demonstrates that a progressive asymmetric ICRS with variable thickness and base width has the potential to specifically correct optical aberrations, which are predominant in asymmetric KC phenotypes. The next generation of ICRSs for clinical application could aim at not only correcting astigmatism but also aberrations such as horizontal/vertical coma and other higher-order aberrations. In this context, the current modelling study permits a more objective evaluation of the optomechanical mechanisms determining the surgical outcomes than a clinical study that has to deal with further confounding factors. The results presented here may serve to guide clinicians in the selection of ICRS, even though there remains future research to be completed in this area before realistic modelling accounting for post-operative tissue remodelling effects becomes possible.

## Figures and Tables

**Table 1:** Material properties and thicknesses of the different parts of the model. Yeoh material constants C1, C2, C3; d = first incompressibility parameter; th = thickness.

		C1	C2	C3	d	th
healthy	anterior	35.5 kPa	3.2 kPa	1.9 kPa	10^−5^ Pa	385 µm
	posterior	32.0 kPa	2.9 kPa	1.7 kPa	10^−5^ Pa	165 µm
KC region1	anterior	24.9 kPa	2.2 kPa	1.3 kPa	10^−5^ Pa	270 µm
	posterior	22.4 kPa	2.0 kPa	1.2 kPa	10^−5^ Pa	116 µm
KC region 2	anterior	10.7 kPa	1.0 kPa	0.57 kPa	10^−5^ Pa	193 µm
	posterior	9.6 kPa	0.86 kPa	0.51 kPa	10^−5^ Pa	83 µm
sclera	-	0.8 MPa	56.1 MPa	2332 MPa	10^−5^ Pa	1000 µm
		E	ρ	ν		
ICRS	-	3.3 GPa	1062 kg/m^3^	0.40		variable

**Table 2:** Bi-conic curvature assessment of different ICRS designs in a simulated generic healthy and keratoconic cornea. asym = progressive asymmetric ICRS; sym = average symmetric ICRS; symMin and symMax = symmetric ICRS with the minimal and maximal cross-section of the progressive asymmetric ICRS; asymTH = asymmetric ICRS with thickness-only variation; asymW = asymmetric ICRS with width-only variation; Rx and Ry = horizontal and vertical radius of curvature; ΔAL = change in axial length of the eye globe; Qx and Qy = horizontal and vertical asphericity; Δdpt = overall dioptric change of the eye considering changes in the anterior, posterior surface, and axial length.

ANTERIOR								
(mm)	Rx	Ry	∆Rx	∆Ry	∆AL	Qx	Qy	∆dpt
healthy	7.37	7.62	-	-	-	−0.25	−0.10	-
healthy asym	8.05	7.95	0.68	0.33	−0.04	1.21	−2.07	−3.17
healthy sym	7.96	8.14	0.59	0.52	−0.04	−0.13	−0.22	−3.44
healthy symMax	8.36	8.35	0.99	0.73	−0.04	0.98	−1.40	−5.14
healthy symMin	7.77	7.87	0.40	0.25	−0.03	−0.18	−0.17	−2.09
healthy asymW	7.97	8.14	0.60	0.52	−0.04	−0.15	−0.21	−3.47
healthy asymTH	7.96	8.13	0.59	0.50	−0.04	0.65	−0.94	−3.40
KC	6.15	7.24	-	-	0.11	−1.55	1.33	-
KC asym	5.88	7.72	−0.26	0.48	−0.04	−3.83	2.19	−0.89
KC sym	6.34	7.61	0.19	0.36	−0.04	−2.17	1.76	−2.23
KC_symMax	6.89	7.61	0.75	0.36	−0.05	−0.19	−0.18	−4.29
KC_symMin	6.20	6.61	0.05	−0.63	−0.04	−1.76	−2.54	2.52
KC_asymW	6.44	7.57	0.29	0.33	−0.04	−1.63	1.53	−2.49
KC_asymTH	6.44	6.79	0.30	−0.46	−0.04	−0.01	−3.77	0.68
POSTERIOR								
healthy	7.91	7.97	-	-	-	0.63	0.37	-
healthy asym	8.35	8.70	0.99	1.08	-	5.18	7.85	0.39
healthy sym	7.17	7.40	−0.20	−0.22	-	−1.39	1.03	−0.51
healthy symMax	8.74	8.69	1.37	1.07	-	8.16	9.08	0.50
healthy symMin	7.51	7.45	0.14	−0.17	-	−0.19	−0.20	−0.35
healthy asymW	7.18	7.37	−0.19	−0.25	-	−1.48	1.08	−0.52
healthy asymTH	7.10	7.46	−0.26	−0.17	-	−1.53	1.10	−0.52
KC	6.18	6.81	-	-	-	−1.13	−1.02	-
KC asym	6.23	7.55	0.08	0.31	-	1.11	7.05	0.40
KC sym	6.34	7.71	0.19	0.47	-	0.66	7.32	0.52
KC_symMax	6.54	7.58	0.40	0.33	-	2.88	7.59	0.56
KC_symMin	5.86	7.18	−0.28	−0.07	-	−2.37	4.01	0.03
KC_asymW	6.48	7.71	0.34	0.47	-	1.42	7.36	0.59
KC_asymTH	5.29	6.35	−0.86	−0.89	-	−2.56	2.42	−0.80

**Table 3:** Changes in Zernike polynomials after ICRS implantation. Changes are computed with respect to the pre-op condition, except pre-op KC, which shows the change with respect to the healthy corneal model.

Zernike Coefficient	∆ Healthy						∆ KC							Description
	asym	sym	symMax	symMin	varWidth	varTH	pre-op	asym	sym	symMax	symMin	varWidth	varTH	
1	5.11	5.52	2.85	7.69	5.54	5.10	14.25	1.37	−2.04	−4.19	−2.04	−1.74	4.77	vertical tilt
2	−3.81	−0.73	−0.13	−1.16	0.46	−5.03	7.57	−9.17	−11.35	−10.87	−11.35	−9.86	−4.82	horizontal tilt
3	1.37	−0.12	−0.27	−0.03	−0.48	1.72	2.60	1.28	−0.19	−0.45	−0.19	−0.55	1.51	oblique primary astigmatism
4	1.18	1.21	2.24	0.34	1.23	1.15	−4.64	2.90	2.75	3.95	2.75	2.83	3.07	defocus
5	−0.16	0.40	−1.18	1.29	0.48	−0.37	1.61	−1.41	−1.57	−3.35	−1.57	−1.28	−1.01	vertical/horizontal primary astigmatism
6	0.08	0.01	−0.40	0.31	0.02	0.10	−0.25	−0.48	−0.51	−1.01	−0.51	−0.41	−0.47	vertical trefoil
7	−1.89	−1.81	−2.72	−0.99	−1.86	−1.84	5.29	−3.42	−3.13	−4.31	−3.13	−3.22	−3.38	vertical coma
8	−0.28	0.28	0.42	0.16	0.05	−0.06	2.37	−0.67	0.36	0.44	0.36	0.00	−0.62	horizontal coma
9	−0.21	−0.06	0.20	−0.28	0.00	−0.29	1.42	−0.54	−0.08	0.05	−0.08	−0.04	−0.71	oblique trefoil
10	0.25	−0.16	−0.31	−0.05	−0.20	0.28	−0.40	0.63	−0.04	−0.03	−0.04	−0.01	0.75	oblique quadrafoil
11	0.21	−0.06	−0.07	−0.04	0.08	0.06	−2.49	0.48	−0.09	0.08	−0.09	0.13	0.50	oblique secondary astigmatism
12	0.86	0.85	1.01	0.65	0.86	0.85	1.68	1.38	0.83	1.10	0.83	0.92	1.85	primary spherical
13	−0.17	−0.13	−0.46	0.07	−0.15	−0.17	1.63	−0.77	−0.90	−1.30	−0.90	−0.84	−0.60	vertical secondary astigmatism
14	−0.49	−0.26	−0.42	−0.13	−0.26	−0.49	−0.46	−0.55	−0.48	−0.55	−0.48	−0.43	−0.53	vertical quadrafoil
∑ low order	−20.77	−21.67	−22.36	−21.56	−21.92	−20.66	−22.59	−20.81	−22.59	−23.43	−22.59	−22.59	−20.02	
∑ high order	3.16	3.47	2.06	4.52	3.37	3.26	9.58	9.69	9.58	8.08	9.58	9.70	10.41	

## Data Availability

Data sharing not applicable.
